# Failed regeneration and inflammation in schizophrenia: two sides of the same coin?

**DOI:** 10.1007/s00702-022-02496-3

**Published:** 2022-04-22

**Authors:** Peter Falkai, Andrea Schmitt

**Affiliations:** 1 Department of Psychiatry and Psychotherapy, University Hospital, LMU Munich, Nussbaumstrasse 7, 80336 Munich, Germany; 2 Laboratory of Neuroscience (LIM27), Institute of Psychiatry, University of São Paulo, São Paulo, Brazil

**Keywords:** Schizophrenia, Neurodevelopment, Neurodegeneration, Neuroinflammation, Neuron, Interneuron, Oligodendrocyte

## Abstract

More than 100 years after its conceptual definition as ‘Dementia Praecox’ by Emil Kraepelin, which was changed to schizophrenia by Eugen Bleuler, this is still a serious and debilitating psychiatric illness. The neurodevelopmental hypothesis of schizophrenia, introduced more than 30 years ago, states that schizophrenia is a consequence of failed neurodevelopmental processes leading to a dysfunctional neuronal network forming the basis for a psychosis proneness. Subsequently, significant research efforts were made to prove the neurodevelopmental or the neurodegenerative perspective. This review summarizes key arguments speaking for or against the two hypotheses leading to a concept with both aspects position side by side.

## Introduction

The neurodevelopmental hypothesis of schizophrenia was introduced more than 30 years ago (Weinberger 1986) stating that schizophrenia is a consequence of failed neurodevelopmental processes leading to a dysfunctional neuronal network forming the basis for a psychosis proneness. This will in young adulthood lead to psychotic symptoms under adding up environmental stressors, e.g. ranging from place and season of birth, bullying to cannabis consumption and several others. The neurodegenerative hypothesis on the other hand suggests that schizophrenia is a consequence of a “destructive process of the brain leading to peculiar changes of the psyche” as outlined by E Kraepelin in his books (Kraepelin 1913). This “destructive process” was based on the first histopathological study by A. Alzheimer (1893) describing “loosening of neural elements in the neocortex” of patients. Subsequently, significant research efforts were made to prove the neurodevelopmental or the neurodegenerative perspective. Meanwhile, the neurodevelopmental hypothesis was widened by the “pandysmaturational hypothesis” (Fish 1987), which enlarges the aspects of disturbed neurodevelopment into adulthood and beyond. The neurodegenerative hypothesis on the other hand currently embarked on neuroimmunological mechanisms based on innovative new technical advances in research.

## Long-term course and functional outcome

**Fig. 1:**
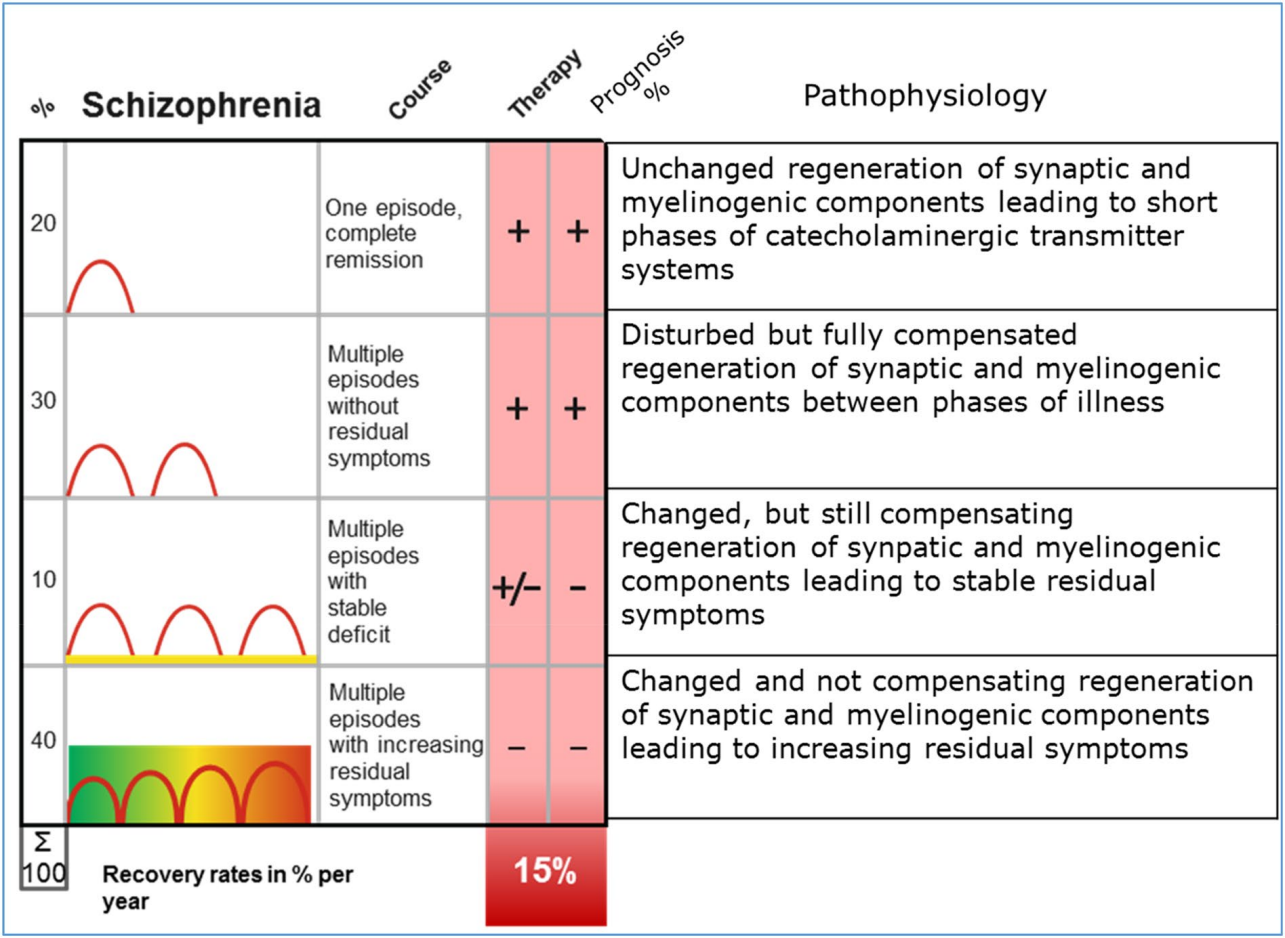
Stratifying Schizophrenia based on its long-term course (Häfner 2003)

There is a large number of studies evaluating the long-term course of schizophrenia, which are summarized in several meta-analyses (Lang et al. 2001; Hasan et al. 2013]. As summarized in Fig. 1 below, about 50% of the patients have no or very little residual symptoms following the first manifestation of the illness or the relapsing course (two upper rows). The other 50% of patients will, however, have residual symptoms (as depicted in yellow or green to red in the lower two rows), especially negative symptoms and cognitive dysfunction, leading to a significant disturbance of their daily functioning. Taken together, only 20% of patients are able to find a job on the primary labour market and only about 30% can keep up a stable long-term relationship (Häfner 2003).

Following the transition from at risk mental or prodromal states of psychosis to the first episode illness, negative symptoms and cognitive dysfunction develop early in the course and remain stable even after 10 years of follow up and are very difficult to treat (Hoff et al. 2005). A substantial proportion of patients reveal a step-like decrease of cognitive functioning, which they have difficulties to climb up again. This gap remains somewhat stable in comparison to control groups where at each follow-up session the test performance improves as the contents of the tests are learned step-by-step (Hoff et al. 1999; DeLisi and Hoff 2005). Beside the step-like decrease of cognitive functioning and negative symptoms, there is very little evidence for a further decline over time for the majority of patients. An exception can be made for a subgroup of patients often named “Kraepelinian schizophrenics” (Keefe et al. 1996). They possibly represent a subgroup with a degenerative course resembling patients with fronto-temporal dementia (FTLD) as has recently been described (Koutsouleris 2021). Even if it is a small subgroup once replicated it would possibly allow specific interventions only working here and not in the majority of patients. The attempt to identify good and bad outcome schizophrenia has a long tradition, e.g. Type I and II (Crow 1985), primary (deficit state) and secondary negative syndrome (Carpenter and Kirkpatrick 1988) and has certainly raised attention to sub-syndromes to find better treatment options for them (Ventura et al. 2019).

## Structural brain-imaging variables and illness course

Structural brain imaging parameters are more stable over time than functional measures or other biological variables. They are less likely representing a functional state, but are less vulnerable to short-term factors like changes in physical conditions or the mental state. Taken together, long-term follow-up MRI studies show a decrease of the brain volumes over time, which is influenced by cumulative antipsychotic exposure (Vita et al. 2015). This is more pronounced under antipsychotics like Haloperidol and to a lesser extent present under Olanzapin or Risperidone (Lieberman et al. 2003). Remarkably, this brain volume reduction is correlated with worse functional outcome (Haren et al. 2003) and seems to represent the underlying neural substrate of the long-term outcome in schizophrenia.

## Cellular elements contributing to the brain volume reduction

What does the volume reduction mentioned in the last paragraph mean? Brain volume reduction is usually associated with neuronal loss and permanent loss of function as part of a neurodegenerative process. This association is too simple, as the brain volume consists of different elements like neurons, glial cells and neuropil. The neuropil itself contains synaptic, axonal and dendritic components. Therefore, a volume reduction can have several reasons. Focussing on the hippocampal formation, one of the key regions involved in volume change in schizophrenia (Erp et al. 2016), stereologically based studies do not show a significant neuronal loss in its sub-segments. Astroglial and interneuronal numbers are unchanged as well but oligodendroglia was reduced in the CA4 subregion (Schmitt et al. 2009). The latter finding was replicated recently in a completely different brain sample using unbiased stereology (Schmitt et al. 2021). Searching for the neural substrate of plasticity related changes due to exercise in the hippocampal formation (Pajonk et al. 2010; Papiol et al. 2017), oligodendroglial precursors and radial glia was connected to the genetic vulnerability to develop schizophrenia (Papiol et al. 2019). Using iPSCs from patients with schizophrenia this finding could be replicated (Skene et al. 2018).

Beside myelin-associated pathology there is consolidated evidence that in schizophrenia synaptic proteins are changed and/or diminished with a focus on fronto-temporal regions (Berdenis van Berlekom et al. 2020) Synaptic changes in the hippocampus seem to be connected with neurodevelopmental abnormalities in the entorhinal cortex (Falkai et al. 2000) supporting the “pandysmaturational hypothesis” , stating that disturbed neurodevelopment relates to all phases of normal development until adulthood and beyond.

## Underlying mechanisms of cellular changes

In a comprehensive meta-analysis on glial cells in schizophrenia, it was found that microglia was increased/activated relating to an acute inflammatory response (Marques et al. 2019). There is a long history of studies searching for bacterial, viral or fungal elements causing inflammatory reactions leading to neural changes underlying the pathophysiology of schizophrenia. None of them has led to replicated candidates relating to the inflammatory response, though it seems clear that the activation of the immune system in the sense of a low-grade inflammation is a well-replicated finding in schizophrenia (Marques et al. 2019; Torrey and Yolken 2019). The related post-mortem literature points to local processes not crossing the blood–brain barrier and can therefore be qualified as an activation of the immune system due to any unspecific insult (Kesteren et al. 2017). Therefore, the activation of the immune system in schizophrenia is part of the plastic response, where the brain and its neural elements try to compensate for processes leading to psychotic symptoms including negative symptoms and cognitive dysfunction in a about half of the patients (see Fig. 1). Depending on how successful the compensation works this will leading to different outcomes (see Fig. 1 from top to bottom row). The pathophysiological process starts with disturbed synaptic and/or myelin-associated mechanisms leading to reduced functioning in neuronal and glial elements, which are most heavily involved in brain activity like interneurons or oligodendroglia. In schizophrenia the relationship of interneuron with oligodendrocyte pathology is unknown. Parvalbuminergic interneurons are ensheathed by myelinating oligodendrocytes and their fast-spiking and very high tonic activity may require the function of myelin in supporting the high-energy demands. Glycolytic oligodendrocytes are known to deliver lactate to axons, thereby providing energetic support of the axonal intermediate metabolism. The concept of metabolic coupling of myelin and axons is an important new development in neuroscience, but it remains to be established if all types of neurons, specifically interneurons need this metabolic support (Schmitt 2019). In summary, understanding molecular processes leading to synaptic and/or myelin-associated pathology and the interplay between neurons and oligodendroglia should be key to understand the pathophysiology of schizophrenia.

## References

[CR1] Alzheimer A (1893). Neuere Arbeiten über die Dementia senilis [recent works on senile dementia]. Monatsschr Psychiatr Neurol.

[CR2] Berdenis van Berlekom A, Muflihah C, Snijders G, MacGillavry HD, Middeldorp J, Hol EM, Kahn RS, de Witte LD (2020). Synapse pathology in schizophrenia a meta-analysis of postsynaptic elements in postmortem brain studies. Schizophr Bull.

[CR3] Carpenter WT, Kirkpatrick B (1988). The heterogeneity of the long-term course of schizophrenia. Schizophr Bull.

[CR4] Crow TJ (1985). The two-syndrome concept: origins and current status. Schizophr Bull.

[CR5] DeLisi LE, Hoff AL (2005). Failure to find progressive temporal lobe volume decreases 10 years subsequent to a first episode of schizophrenia. Psychiatry Res.

[CR6] Falkai P, Schneider-Axmann T, Honer WG (2000). Entorhinal cortex pre-alpha cell clusters in schizophrenia: quantitative evidence of a developmental abnormality. Biol Psychiatry.

[CR7] Fish B (1987). Infant predictors of the longitudinal course of schizophrenic development. Schizophr Bull.

[CR8] Häfner H, Heiden de W, Hirsch R, Weinberger D (2003). Course and outcome of schizophrenia. schizophrenia.

[CR9] Hasan A (2013). World Federation of Societies of Biological Psychiatry (WFSBP) guidelines for biological treatment of schizophrenia, part 2: update 2012 on the long-term treatment of schizophrenia and management of antipsychotic-induced side effects. World J Biol Psychiatry.

[CR10] Hoff AL, Sakuma M, Wieneke M, Horon R, Kushner M, DeLisi LE (1999). Longitudinal neuropsychological follow-up study of patients with first-episode schizophrenia. Am J Psychiatry.

[CR11] Hoff AL, Svetina C, Shields G, Stewart J, DeLisi LE (2005). Ten year longitudinal study of neuropsychological functioning subsequent to a first episode of schizophrenia. Schizophr Res.

[CR12] Keefe RS, Frescka E, Apter SH, Davidson M, Macaluso JM, Hirschowitz J, Davis KL (1996). Clinical characteristics of Kraepelinian schizophrenia: replication and extension of previous findings. Am J Psychiatry.

[CR13] Koutsouleris N et al. 2021. Revisiting ‘Dementia Praecox’ - Comparing Frontotemporal Dementia and Psychosis using Multi-modal, Multi-cohort Machine Learning. Jama Psychiatry; accepted.

[CR14] Kraepelin E (1913). Lehrbuch der Psychiatrie [Textbook of Psychiatry].

[CR15] Lang DJ, Kopala LC, Vandorpe RA, Rui Q, Smith GN, Goghari VM, Honer WG (2001). An MRI study of basal ganglia volumes in first-episode schizophrenia patients treated with risperidone. Am J Psychiatry.

[CR16] Lieberman JA, Tollefson G, Tohen M, Green AI, Gur RE, Kahn R, McEvoy J, Perkins D, Sharma T, Zipursky R, Wei H, Hamer RM, HGDH Study Group (2003). Comparative efficacy and safety of atypical and conventional antipsychotic drugs in first-episode psychosis: a randomized, double-blind trial of olanzapine versus haloperidol. Am J Psychiatry.

[CR17] Marques TR, Ashok AH, Pillinger T, Veronese M, Turkheimer FE, Dazzan P, Sommer IEC, Howes OD (2019). Neuroinflammation in schizophrenia: meta-analysis of in vivo microglial imaging studies. Psychol Med.

[CR18] Pajonk FG, Wobrock T, Gruber O, Scherk H, Berner D, Kaizl I (2010). Hippocampal plasticity in response to exercise in schizophrenia. Arch Gen Psychiatry.

[CR19] Papiol S, Popovic D, Keeser D, Hasan A, Schneider-Axmann T, Degenhardt F, Rossner MJ, Bickeböller H, Schmitt A, Falkai P, Malchow B (2017). Polygenic risk has an impact on the structural plasticity of hippocampal subfields during aerobic exercise combined with cognitive remediation in multi-episode schizophrenia. Transl Psychiatry.

[CR20] Papiol S, Keeser D, Hasan A, Schneider-Axmann T, Raabe F, Degenhardt F, Rossner MJ, Bickeböller H, Cantuti-Castelvetri L, Simons M, Wobrock T, Schmitt A, Malchow B, Falkai P (2019). Polygenic burden associated to oligodendrocyte precursor cells and radial glia influences the hippocampal volume changes induced by aerobic exercise in schizophrenia patients. Transl Psychiatry.

[CR21] Schmitt A, Steyskal C, Bernstein HG, Schneider-Axmann T, Parlapani E, Schaeffer EL, Gattaz WF, Bogerts B, Schmitz C, Falkai P (2009). Stereologic investigation of the posterior part of the hippocampus in schizophrenia. Acta Neuropathol.

[CR22] Schmitt A, Simons M, Cantuti-Castelvetri L, Falkai P (2019). A new role for oligodendrocytes and myelination in schizophrenia and affective disorders?. Eur Arch Psychiatry Clin Neurosci.

[CR23] Schmitt A, Tatsch L, Vollhardt A, Heinsen H, Hof PR, Falkai P, Schmitz C. Decreased number of oligodendrocytes in hippocampal subfield CA4 in schizophrenia. Preparation of manuscript in progress; 2021 unpublished data. 10.3390/cells11203242 PMC9600243 36291109

[CR24] Skene NG, Bryois J, Bakken TE, Breen G, Crowley JJ, Gaspar HA, Giusti-Rodriguez P, Hodge RD, Miller JA, Muñoz-Manchado AB, O'Donovan MC, Owen MJ, Pardiñas AF, Ryge J, Walters JTR, Linnarsson S, Lein ES, Sullivan PF, Hjerling-Leffler J, Major Depressive Disorder Working Group of the Psychiatric Genomics Consortium (2018). Genetic identification of brain cell types underlying schizophrenia. Nat Genet.

[CR25] Torrey EF, Yolken RH (2019). Schizophrenia as a pseudogenetic disease: a call for more gene-environmental studies. Psychiatry Res.

[CR26] van Erp TG, Hibar DP, Rasmussen JM, Glahn DC, Pearlson GD, Andreassen OA, Agartz I, Westlye LT, Haukvik UK, Dale AM, Melle I, Hartberg CB, Gruber O, Kraemer B, Zilles D, Donohoe G, Kelly S, McDonald C, Morris DW, Cannon DM, Corvin A, Machielsen MW, Koenders L, de Haan L, Veltman DJ, Satterthwaite TD, Wolf DH, Gur RC, Gur RE, Potkin SG, Mathalon DH, Mueller BA, Preda A, Macciardi F, Ehrlich S, Walton E, Hass J, Calhoun VD, Bockholt HJ, Sponheim SR, Shoemaker JM, van Haren NE, Hulshoff Pol HE, Ophoff RA, Kahn RS, Roiz-Santiañez R, Crespo-Facorro B, Wang L, Alpert KI, Jönsson EG, Dimitrova R, Bois C, Whalley HC, McIntosh AM, Lawrie SM, Hashimoto R, Thompson PM, Turner JA (2016). Subcortical brain volume abnormalities in 2028 individuals with schizophrenia and 2540 healthy controls via the ENIGMA consortium. Mol Psychiatry.

[CR27] van Haren NE, Cahn W, Hulshoff Pol HE, Schnack HG, Caspers E, Lemstra A, Sitskoorn MM, Wiersma D, van den Bosch RJ, Dingemans PM, Schene AH, Kahn RS (2003). Brain volumes as predictor of outcome in recent-onset schizophrenia: a multi-center MRI study. Schizophr Res.

[CR28] Van Kesteren CF, Gremmels H, de Witte LD, Hol EM, Van Gool AR, Falkai PG, Kahn RS, Sommer IE (2017). Immune involvement in the pathogenesis of schizophrenia: a meta-analysis on postmortem brain studies. Transl Psychiatry.

[CR29] Ventura J, Subotnik KL, Gretchen-Doorly D, Casaus L, Boucher M, Medalia A, Bell MD, Hellemann GS, Nuechterlein KH (2019). Cognitive remediation can improve negative symptoms and social functioning in first-episode schizophrenia: a randomized controlled trial. Schizophr Res.

[CR30] Vita A, De Peri L, Deste G, Barlati S, Sacchetti E (2015). The effect of antipsychotic treatment on cortical gray matter changes in schizophrenia: does the class matter? A Meta-analysis and meta-regression of longitudinal magnetic resonance imaging studies. Biol Psychiatry.

[CR31] Weinberger DR, Nasrallah RA, Weinberger DR (1986). The pathogenesis of schizophrenia: a neurodevelopmental theory. The Neurology of Schizophrenia, 387–405.

